# Commentary on the T1D exchange quality improvement collaborative learning session November 2023 abstracts

**DOI:** 10.1111/1753-0407.13496

**Published:** 2024-01-17

**Authors:** Shideh Majidi, Shivani Agarwal, Nicole Rioles, Robert Rapaport, Osagie Ebekozien, for the T1DX‐QI Collaborative

**Affiliations:** ^1^ Children's National Hospital Washington District of Columbia USA; ^2^ Fleischer Institute for Diabetes and Metabolism, Albert Einstein College of Medicine, Montefiore Medical Center Bronx New York USA; ^3^ NY‐Regional Center for Diabetes Translational Research, Albert Einstein College of Medicine, Montefiore Medical Center Bronx New York USA; ^4^ T1D Exchange Boston Massachusetts USA; ^5^ Mount Sinai Kravis Children's Hospital, Icahn School of Medicine New York New York USA

**Keywords:** psychosocial, quality improvement, remote patient monitoring, technology, type 1 diabetes

## 1 INTRODUCTION

The T1D Exchange Quality Improvement Collaborative (T1DX‐QI), established in 2016, aims to refine best practices, emphasize quality of care, and improve diabetes outcomes across the country in those with type 1 diabetes (T1D).^1^ T1DX‐QI includes over 55 diabetes centers, including both adult and pediatric diabetes centers, allowing it to investigate and improve diabetes care for over 85 000 people. Over the past 7 years, it has been on the forefront of collaborative care and using real‐world data to improve diabetes care and outcomes.

**FIGURE 1:**
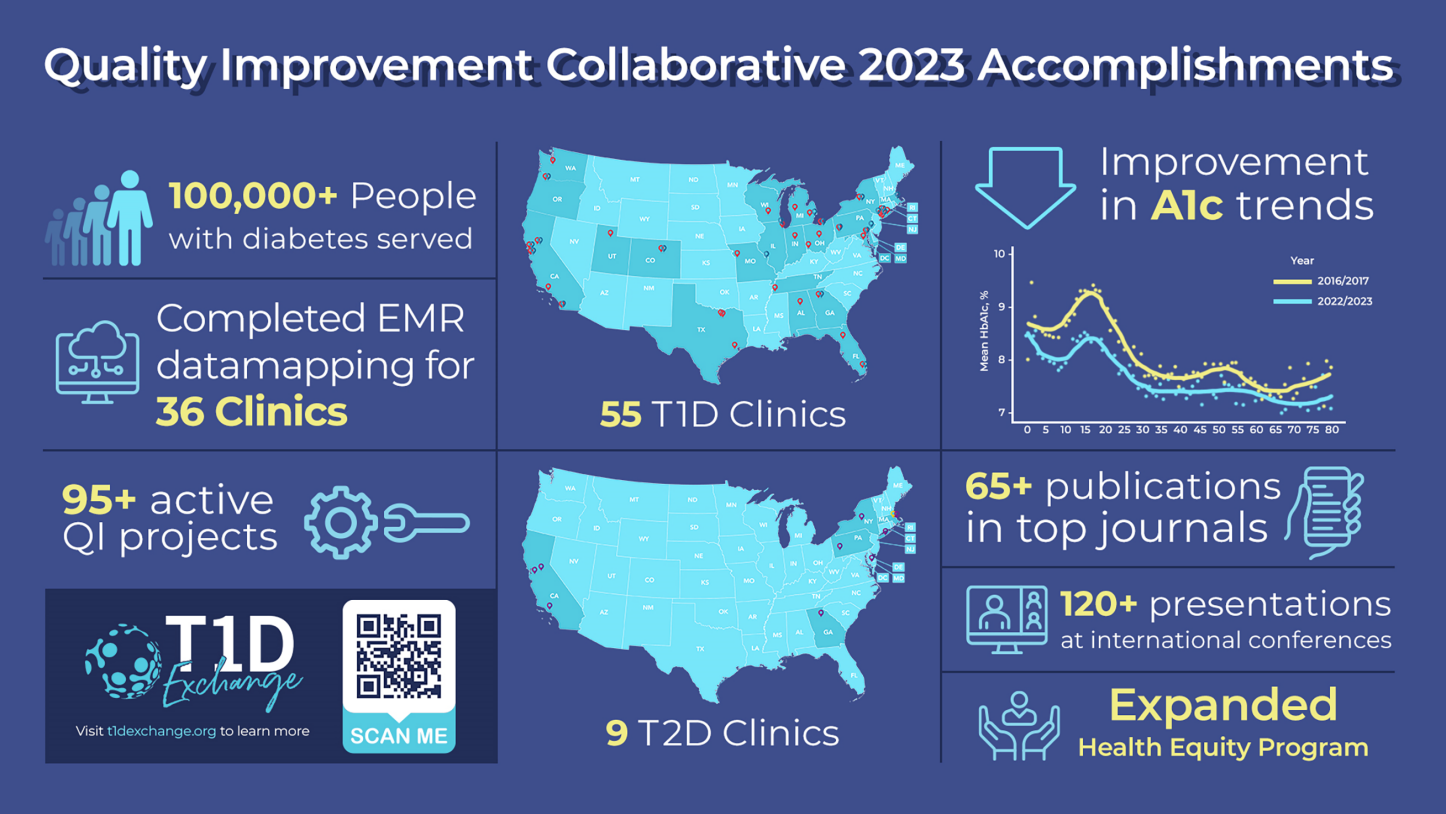
T1D Exchange Quality Improvement Collaborative (T1DX‐QI 2023) accomplishments. EMR, electronic medical record; T1D, type 1 diabetes; T2D, type 2 diabetes.

The network has had a positive impact on the community, as it continues to bring together centers to support real‐world improvement and outcomes, achieving great results. Figure 1 shows the impact of the T1DX‐QI group in 2023. The November 2023 conference, similar to previous years, encompasses a wide range of topics in diabetes care and practice improvement, with a focus on psychosocial aspects of care and improving technology advancement and access.

Behavioral health abstracts included screening of eating disorders^2^ and anxiety.^3^ Gillis et al^3^ looked at how anxiety affects hemoglobin A1C (A1C) across T1DX‐QI sites and found a greater percentage with anxiety have an A1C >9% and that females more than males have elevated anxiety levels. Adams et al^4^ and Weinstock et al^5^ focused on implementation and outcomes of social determinants of health (SDOH) screening in pediatric and adult diabetes centers. Weinstock et al^5^ found those who screened positive were more likely Black and had more than one need to be addressed. The highest need identified on SDOH screening was food insecurity. Ruiz et al^6^ implemented food insecurity screening in their diabetes center and successfully increased the percentage of patients who were screened and received resources.

Several abstracts focused on identifying barriers to technology use and improving use in T1D, particularly technology uptake and equity. In terms of smart insulin pens (SIP), Miyazaki et al^7^ found poor uptake related to copay requirement, lack of samples, and poor responsiveness when trainer reached out to families. Figueredo et al^8^ found significant barriers to patients saving and sharing SIP data, which affects providers ability to make management decisions. Regarding uptake and initiation of continuous glucose monitors (CGM), Milosavljevic et al^9^ found Hispanic race‐ethnicity has the lowest CGM prescription rate in their center. Meanwhile, Perkins et al^10^ created an electronic medical record tool to track CGM uptake from initial prescribing through obtaining and receiving education on CGM in order to centralize and more closely monitor patients actually obtaining and starting the device. Several diabetes centers focused on increasing insulin pump uptake and use. Miyazaki et al^11^ found that by reducing stricter requirements for insulin dose optimization prior to discussion of starting an insulin pump, patients with public insurance were 5.6 times as likely to initiate an insulin pump. Ogburn et al^12^ focused on decreasing time to insulin pump start and found that virtual pump class or phone follow‐up led to shorter time to insulin pump start. Smego et al^13^ found that a pump introduction self‐learning packet and virtual pump classes resulted in an increase in insulin pump starts. Coppedge et al^14^ focused on improving use of hybrid closed loop systems (HCLS) by increasing education on HCLS and having clinic staff transition patients to HCLS, resulting in overall clinic time in range increase and A1C improvement. Focusing on health disparities,^15^, ^16^ Jones et al^15^ implemented several targeted strategies to decrease disparities and improve CGM use. Their multifaceted approach resulted in significant increases in CGM use in the Black population and in those with public insurance, with associated decreases in A1C levels. Cymbaluk et al^16^ also implemented a multifaceted approach to decrease disparities in insulin pump use and found a subsequent increase of 12% in those with public insurance utilizing an insulin pump.

Remote patient monitoring (RPM), the use of connected electronic tools to record medical data that are then reviewed remotely by a provider, has become of greater interest since the increase in telemedicine use during the COVID‐19 pandemic. RPM was the focus of two clinics' efforts to improve diabetes care.^17^, ^18^, ^19^ Petty et al^17^ found that 73.3% of patients with A1C ≥9% participating in RPM between visits had an improvement in A1C levels, with a median change of −1.0%. DeSalvo et al^18^ implemented an RPM program both in newly diagnosed and established patients who are at moderate‐to‐high risk for diabetes ketoacidosis. Improvement has been seen in self‐management habits.^19^

With the approval of teplizumab‐mzwv, there has been greater interest and need for clinical protocols around early staging and monitoring of T1D. To this end, Simmons et al^20^ demonstrate identification of early T1D patients and implementation of a clinic focused on those with early stages of T1D.

## 2 SUMMARY

The 2023 abstracts show continued strong efforts from the T1DX‐QI Collaborative to focus on improving real‐world diabetes care in real time and remaining on the cutting edge of new treatments and approaches to diabetes management. Future directions include continuing our focus in the three key areas of (a) improving overall health outcomes; (b) reducing complications, including the support of cardiovascular health; and (c) and closing health equity gaps for people living with T1D.

## AUTHOR CONTRIBUTIONS

Osagie Ebekozien conceptualized the manuscript. Shideh Majidi wrote the manuscript. Shivani Agarwal, Nicole Rioles, and Robert Rapaport reviewed/edited and approved the final versions of the manuscript. Osagie Ebekozien and Nicole Rioles are the guarantors of this work.

## FUNDING INFORMATION

Leona M. and Harry B. Helmsley Charitable Trust.

## CONFLICT OF INTEREST STATEMENT

Shideh Majidi and Nicole Rioles have no disclosures. Robert Rapaport is an Editorial Board member of Journal of Diabetes and a co‐author of this article. To minimize bias, they were excluded from all editorial decision‐making related to the acceptance of this article for publication. Shivani Agarwal works as a health care disparities advisor for Medtronic Inc. and Beta Bionics. Osagie Ebekozien is a member of the Medtronic Diabetes Health Equity Advisory Board. Osagie Ebekozien is the Principal Investigator on investigator‐initiated projects funded by Medtronic Diabetes, Eli Lilly, and Dexcom. All compensation is direct to his organization T1D Exchange.
